# Epidemiology of invasive aspergillosis in critically ill patients: clinical presentation, underlying conditions, and outcomes

**DOI:** 10.1186/s13054-014-0722-7

**Published:** 2015-01-12

**Authors:** Fabio Silvio Taccone, Anne-Marie Van den Abeele, Pierre Bulpa, Benoit Misset, Wouter Meersseman, Teresa Cardoso, José-Artur Paiva, Miguel Blasco-Navalpotro, Emmanuel De Laere, George Dimopoulos, Jordi Rello, Dirk Vogelaers, Stijn I Blot, on behalf of the AspICU Study Investigators

**Affiliations:** Department of Intensive Care Erasme Hospital, Free University of Brussels, Route de Lennik 808, 1070 Brussels, Belgium; Department of Microbiology General Hospital St. Lucas, Groenebriel 1, 9000 Ghent, Belgium; Department of Intensive Care Mont-Godinne University Hospital, Catholic University of Louvain, Avenue G.Thérasse 1, 5530 Yvoir, Belgium; Department of Intensive Care Foundation Hospital Saint-Joseph, Paris-Descartes University, 185 Rue Raymond Losserand, 75014 Paris, France; Medical Intensive Care Unit, University Hospital Leuven, Herestraat 49, 3000 Leuven, Belgium; Department of Intensive Care, Santo Antonio Hospital, Largo Prof. Abel Salazar, 4099-001 Porto, Portugal; Department of Emergency and Intensive Care, Hospital Centre S. Joao and University of Porto Medical School Alameda Professor Hernâni Monteiro, 4200-319 Porto, Portugal; Department of Intensive Care, University Hospital Severo Ochoa, Avenida de Orellana, s/n 28911 Leganés Madrid, Spain; Department of Microbiology, General Hospital Delta, Brugsesteenweg 90, 8800 Roeselare, Belgium; Department of Critical Care Medicine, Attikon University Hospital, University of Athens Medical School, 1 Rimini Street, Haidari, 124 62 Athens, Greece; Hospital Universitari Vall d’Hebron, Vall D’Hebron, Institute of Research, CIBERES, Autonomous University of Barcelona, Passeig Vall d’Hebron, 119-129, 08035 Barcelona, Spain; Department of Internal Medicine Faculty of Medicine & Health Science, Ghent University, De Pintelaan 185, 9000 Ghent, Belgium; Burns, Trauma, and Critical Care Research Centre, The University of Queensland, Butterfield Street, Herston (Brisbane), 4006 Queensland, Australia

## Abstract

**Introduction:**

Invasive aspergillosis (IA) is a fungal infection that particularly affects immunocompromised hosts. Recently, several studies have indicated a high incidence of IA in intensive care unit (ICU) patients. However, few data are available on the epidemiology and outcome of patients with IA in this setting.

**Methods:**

An observational study including all patients with a positive *Aspergillus* culture during ICU stay was performed in 30 ICUs in 8 countries. Cases were classified as proven IA, putative IA or *Aspergillus* colonization according to recently validated criteria. Demographic, microbiologic and diagnostic data were collected. Outcome was recorded 12 weeks after *Aspergillus* isolation.

**Results:**

A total of 563 patients were included, of whom 266 were colonized (47%), 203 had putative IA (36%) and 94 had proven IA (17%). The lung was the most frequent site of infection (94%), and *Aspergillus fumigatus* the most commonly isolated species (92%). Patients with IA had higher incidences of cancer and organ transplantation than those with colonization. Compared with other patients, they were more frequently diagnosed with sepsis on ICU admission and more frequently received vasopressors and renal replacement therapy (RRT) during the ICU stay. Mortality was 38% among colonized patients, 67% in those with putative IA and 79% in those with proven IA (*P* < 0.001). Independent risk factors for death among patients with IA included older age, history of bone marrow transplantation, and mechanical ventilation, RRT and higher Sequential Organ Failure Assessment score at diagnosis.

**Conclusions:**

IA among critically ill patients is associated with high mortality. Patients diagnosed with proven or putative IA had greater severity of illness and more frequently needed organ support than those with *Aspergillus* spp colonization.

## Introduction

Invasive aspergillosis (IA) is a serious opportunistic infection that mainly affects immunocompromised patients, such as those with prolonged neutropenia and cancer [1]. As such, most research on the epidemiology and clinical impact of *Aspergillus* spp infection has been conducted in patients with hematologic malignancies or after stem cell and solid organ transplants [2,3]. However, several reports have shown that *Aspergillus* spp can cause invasive disease in other categories of patients, including those admitted to intensive care units (ICUs) [4-8]. In this setting, clinical diagnosis of IA is a real challenge, as standard diagnostic definitions were developed for, and have been validated only for, patients with cancer or after hematopoietic stem cell transplants and cannot necessarily be extrapolated to critically ill patients, who lack specific host factors as defined by the European Organization for Research and Treatment of Cancer (EORTC) [9].

Although IA has been considered a rare condition among critically ill patients [10-12], recent data indicate that it should be reconsidered as an emerging and devastating infectious disease in this population. Indeed, in an 18-month surveillance program involving 18 Italian ICUs, only 12 cases of IA occurred in 5,561 patients (0.2%), but mortality among these patients was 60% [13]. Also, in another study, researchers reported 7% of patients with IA among whom the mortality rate of 91%. Interestingly, 70% of these patients had no predisposing factors for invasive fungal disease [14]. Moreover, in ICU patients, IA may potentially affect multiple organs and evolve into a disseminated disease, which remains largely underdiagnosed and is associated with poor outcome [15].

In clinical practice, a diagnosis of IA is frequently suspected when *Aspergillus* is isolated from non-sterile body sites, particularly tracheal and bronchial aspirates [16]. However, because *Aspergillus* spp are ubiquitous, one must be cautious in ascribing a pathogenic role to the fungus obtained from these samples. Moreover, the impact of *Aspergillus* spp isolates from respiratory cultures on the occurrence of IA has been studied extensively in immunocompromised patients, but little is known about this effect in other populations, including ICU patients [17,18]. The presence of other risk factors, such as chronic lung and liver disease or general debilitation, may strengthen the likely clinical relevance of a positive *Aspergillus* culture [15]. Nevertheless, invasive diagnostic procedures such as lung biopsy, which are necessary to confirm a diagnosis of *Aspergillus* infection, are often not feasible in patients with severe respiratory insufficiency and critical illness [19-21]. Moreover, non-invasive diagnostic tests, such as galactomannan (GM) determination, have been integrated into a diagnostic algorithm that has been validated only for patients with bone marrow transplant and cancer, so it needs to be further studied in ICU patients [5,22].

The aim of this study was therefore to collect data from a large series of ICU patients with either *Aspergillus* colonization or invasive disease in order to investigate the epidemiology of IA in this population.

## Material and methods

### Patients and settings

We conducted an international, multicenter (*N* = 30 centers) observational study of ICU patients with evidence of either *Aspergillus* colonization or IA (*Asp*ICU Study). All consecutive adult (>18 years) ICU patients with a culture and/or direct examination and/or histopathologic sample positive for *Aspergillus* spp at any site between January 2000 and January 2011 were eligible for inclusion. Patients with a post-mortem diagnosis of IA were also eligible. Data were collected prospectively. However, because we anticipated a relative paucity of patients in whom histopathologic data were available, we accepted patients from historical cohorts (starting from January 2000) on the condition that none of the requested data were missing. Clinical suspicion of IA prior to ICU admission was an exclusion criterion. The study was approved by the local ethics committees and institutional review boards of each participating center (Appendix 1), and, because of the observational nature of the study and the lack of any modification in the general management of these patients, the need for informed consent was waived. A complete and detailed description of the study methodology is reported elsewhere [23].

### Data collection and outcomes

Data were collected from patient medical records and submitted via a web-based registration system [24]. Collected patient data included demographics (age, weight, height and sex), underlying diseases and acute illness severity scores, including the Acute Physiology and Chronic Health Evaluation (APACHE) II score on admission [25] and the Sequential Organ Failure Assessment (SOFA) score [26] on the day of the positive *Aspergillus* culture. Acute respiratory distress syndrome (ARDS) was defined according to the 1994 Consensus Conference criteria [27]. Sepsis was defined according to standard criteria [28]. We also collected clinical data, including signs compatible with invasive fungal disease (that is, refractory or recrudescent fever, pleuritic chest pain or rub, dyspnea, hemoptysis or worsening lung function). Sampling techniques and sites, as well as mycologic test results (including GM measurements and *Aspergillus* PCR, whenever available) to support a diagnosis of IA, were recorded. Test results were interpreted as positive if they met international consensus criteria [5,9,29]. In particular, GM was considered abnormal if the optical density index was >0.5. Organs affected by *Aspergillus* and species identification were recorded. Radiologic data included findings from chest X-rays or computed tomography (CT) scans of involved organs (chest, sinuses, abdomen and central nervous system). Findings on chest CT scans suggestive of invasive fungal disease were defined as “typical” if at least one of the following was present: wedge-shaped lesion, halo or air-crescent sign, lung cavitation or nodule [9].

The date of diagnosis of IA was assigned as the date of the first positive *Aspergillus* culture or as the date of clinical deterioration compatible with fungal disease in cases of postmortem diagnosis. Although the “clinical” diagnosis of IA was reported in the database according to the judgment of the attending physicians, the final diagnosis was obtained using a central adjudication committee for the various diagnostic categories according to (1) the EORTC/Mycosis Study Group (MSG) criteria (proven, probable, possible IA or not classifiable) [9] and (2) “validated” criteria to discriminate *Aspergillus* colonization from IA in critically ill patients (putative or proven IA) (Appendix 2) [23]. Among those patients with putative or proven IA, we separately analyzed those who had EORTC host factors involved in immunosuppression (that is, recent history of neutropenia (<500 neutrophils/mm^3^), receipt of an allogeneic stem cell transplant, prolonged use of corticosteroids at a mean minimum dose of 0.3 mg/kg/day of prednisone equivalent for 13 weeks, treatment with other recognized T cell immunosuppressants and severe inherited immunodeficiency) [9]. Data were also collected on antifungal therapy and its duration as well as outcomes, including length of ICU stay, 12-week survival rate and use of mechanical ventilation and/or renal replacement therapy (RRT) or vasopressor agents, both at the time of the first *Aspergillus*-positive culture and during the ICU stay.

### Statistical analysis

Statistical analyses were performed using the SPSS 18.0 for Windows NT software package (SPSS, Chicago, IL, USA). Descriptive statistics were computed for all study variables. Discrete variables are expressed as counts (percentage) and continuous variables as mean ± standard deviation or median (first to third interquartile range). Differences between the three study groups were assessed using the χ^2^ test, Fisher’s exact test, Student’s *t*-test or the Mann–Whitney *U* test, as appropriate. A time-to-death analysis was performed until 84 days (12 weeks) after the first positive culture for *Aspergillus.* The Kaplan-Meier method with a log-rank test was used to compare the survival curves of the three populations (colonization, putative IA and proven IA) over time. This long-rank test was then adjusted for all clinically relevant variables that could affect outcome (that is, age, sex, medical admission, sepsis on admission, acute respiratory distress syndrome (ARDS) on admission, diagnostic category, APACHE II score on admission, bone marrow transplant, comorbidities, SOFA score on the day of positive *Aspergillus* culture, lung involvement, comorbidities, EORTC criteria for IA, antifungal therapy, vasopressors, mechanical ventilation and RRT). The hazard ratio (HR) for death and 95% confidence interval (CIs) are reported. Multivariable logistic regression analysis with mortality as the dependent variable was performed in patients with putative or proven IA. Only variables associated with a higher risk of 12-week mortality (*P* < 0.2) on a univariate basis were introduced into the multivariate model. Collinearity between variables was excluded prior to modeling. Odds ratios (ORs) with 95% CIs were computed. All tests were two-sided, and a *P*-value <0.05 was considered statistically significant.

## Results

### Characteristics of the study cohort

A total of 563 patients from 30 ICUs in 8 countries (Belgium, France, Brazil, China, Spain, Greece, India and Portugal) were included in the study. The characteristics of the cohort are shown in Table 1. Most patients were medical admissions (*n* = 392, 70%). The most common reasons for ICU admission were respiratory disease (*n* = 222, 39%) and cardiovascular disease (*n* = 147, 26%). The most common comorbid conditions were chronic obstructive pulmonary disease (COPD) (*n* = 174, 31%) and diabetes (*n* = 92, 16%). Fifty-nine (11%) patients were receiving immunosuppressive therapy. Two hundred fifty-six (45%) were receiving corticosteroids, and one hundred sixty-eight (30%) of these patients were on prolonged (>30 days) corticosteroid therapy. Sepsis and ARDS were diagnosed on admission in 222 (39%) and 57 (10%) patients, respectively. Fifty-six patients (10%) had undergone solid organ transplants. Neutropenia was present in 40 patients (6%), but it was prolonged (>10 days) in only 3 patients.

**Table 1:** **Characteristics of the study cohort on intensive care unit admission** ^**a**^

	**All patients (** ***N*** **= 563)**	**Proven IA (** ***n*** **= 94)**	**Putative IA (** ***n*** **= 203)**	**Colonization (** ***n*** **= 266)**
Age, yr	61 ± 17	60 ± 13	62 ± 16	61 ± 18
Male, *n* (%)	341 (61)	54 (57)	127 (63)	160 (60)
BMI, kg/m^2^	24 (21 to 27)	24 (21 to 26)	23 (20 to 27)	25 (22 to 28)
Underlying conditions, *n* (%)				
No underlying disease	76 (14)	4 (4)^b^	11 (5)^b^	61 (23)
COPD	174 (31)	22 (23)^#^	80 (39)^b^	72 (27)
Chronic heart failure	55 (10)	8 (9)	19 (9)	28 (11)
Diabetes	92 (16)	19 (19)	33 (16)	40 (15)
Solid tumor	58 (10)	13 (14)	21 (9)	24 (9)
Hematologic cancer/BMT	48 (8)	15 (16)^b^	31 (15)^b^	6 (2)
Neutropenia	40 (7)	5 (5)^b^	18 (9)^b^	3 (1)
Radiotherapy/chemotherapy	53 (9)	12 (13)^b^	33 (16)^b^	8 (3)
Solid organ transplant	56 (10)	19 (20)^b^	28 (14)^b^	9 (4)
Immunosuppressive drugs	59 (11)	27 (29)^b^^#^	25 (12)^b^	7 (3)
HIV	5 (1)	1 (1)	1 (1)	3 (1)
Liver disease	40 (7)	13 (14)^b^	14 (7)	13 (5)
Chronic hemodialysis	22 (4)	3 (3)	8 (4)	11 (4)
Smoking	88 (16)	15 (16)	28 (14)	45 (17)
Alcohol abuse	54 (10)	9 (10)	16 (8)	29 (11)
Diagnostic categories, *n* (%)				
Medical admission	392 (70)	79 (84)^b,c^	94 (46)^b^	152 (57)
Cardiovascular	147 (26)	29 (30)	49 (24)	69 (26)
Respiratory	222 (39)	38 (41)^b,c^	107 (53)^b^	77 (29)
Neurological	32 (6)	3 (3)^b^	3 (2)^b^	26 (10)
Intoxication	5 (1)	0	1 (1)	4 (2)
Gastrointestinal	37 (7)	6 (7)	13 (6)	18 (7)
Endocrine	18 (3)	8 (9)^b^	10 (5)^b^	0
Post-operative	48 (9)	7 (8)	15 (7)	26 (10)
Trauma	47 (8)	2 (2)^b^	3 (2)^b^	42 (16)
Others	7 (1)	1 (1)	2 (1)	4 (1)
Severity scores and main diagnoses				
APACHE II score on admission	23 (17 to 28)	24 (18 to 28)	25 (17 to 30)	23 (17 to 28)
Sepsis on ICU admission	222 (39)	47 (51)^b^	94 (46)^b^	81 (30)
ARDS on ICU admission	57 (10)	24 (26)^b^^#^	21 (10)^b^	12 (4)
Septic shock, *n* (%)	64 (11)	14 (15)^b^	29 (14)^b^	21 (8)
Traumatic brain injury, *n* (%)	28 (5)	0^b^	1 (1)^b^	27 (10)
Pneumonia, *n* (%)	72 (13)	15 (16)	29 (14)	28 (11)
Other brain injuries, *n* (%)	23 (4)	1 (1)^b^	2 (1)^b^	20 (8)
Acute heart failure/CABG, *n* (%)	39 (7)	3 (3)^b^	6 (3)^b^	30 (11)
Pancreatitis, *n* (%)	7 (1)	1 (1)	1 (1)	5 (1)
Prophylactic antifungals, *n* (%)	32 (6)	8 (9)^b^	19 (9)^b^	5 (2)
Antifungal therapy, *n* (%)	285 (51)	78 (85)^b,c^	147 (72)^b^	60 (22)
Duration of therapy, days^d^	13 (6 to 28)	10 (4 to 24)	12 (7 to 29)	18 (9 to 28)

^#^p < 0.05 vs. Putative IA ^a^APACHE, Acute Physiology and Chronic Health Evaluation; ICU, Intensive care unit; ARDS, Acute respiratory distress syndrome; BMI, Body mass index; BMT, Bone marrow transplant; CABG, Coronary artery bypass graft; COPD, Chronic obstructive pulmonary disease; IA, Invasive aspergillosis; RRT, Renal replacement therapy. ^b^ *P* < 0.05 versus colonization; ^c^ *P* < 0.05 versus putative IA. ^d^Calculated among those patients receiving therapy.

In the total cohort of 563 patients, 94 had proven IA (17%), 203 had putative IA (36%) and 266 were colonized (47%) on the basis of “validated” criteria. It would not have been possible to classify 438 (77%) of the patients if only the EORTC/MSG criteria had been used. *Aspergillus fumigatus* was the most commonly isolated species (*n* = 519, 92%).

### Clinical signs and medical imaging

Clinical and radiologic findings are reported in Table 2. Chest CT was performed in 223 (40%) patients and bronchoalveolar lavage (BAL) in 225 (40%) patients, and serum or BAL GM was measured in 151 (27%) patients. There were significantly more radiologic findings typical of IA on chest CT scans in patients with proven or putative IA than in those with colonization. CT of the sinuses was performed in 24 patients, and 15 of the patients had abnormal scans. Five of the twenty-four patients had sinus cultures positive for *Aspergillus*. All five of these patients had abnormal CT scan findings, but three were diagnosed as colonization and two as putative IA. Abdominal CT was performed in 38 patients, and 24 of these patients had abnormal scans. Three of these thirty-eight patients had positive *Aspergillus* cultures on abdominal samples (two in hepatic samples and one in a peritoneal sample). All 38 patients had abnormal CT scan findings and were diagnosed with proven IA. Cerebral CT was performed in 45 patients, and 30 of these patients had abnormal scans. Nine of the forty-five patients had positive *Aspergillus* cultures in cerebral samples, which were either cerebrospinal fluid (CSF) or derived from brain biopsy. All had abnormal CT scan findings and were diagnosed with proven IA. One additional patient, in whom no cerebral CT was performed, had CSF cultures positive for *Aspergillus*.

**Table 2:** **Clinical, radiologic and microbiologic findings related to** ***Aspergillus*** **diagnosis**

	**All patients (** ***N*** **= 563)**	**Proven IA (** ***n*** **= 94)**	**Putative IA (** ***n*** **= 203)**	**Colonization (** ***n*** **= 266)**
ICU stay before first positive culture, days	4 (1 to 9)	4 (1 to 11)	4 (2 to 10)	4 (2 to 9)
SOFA II score at diagnosis	8 (4 to 12)	11 (7 to 14)^b,c^	9 (6 to 12)^b^	5 (3 to 10)
Clinical signs, *n* (%)				
At least one of the following signs	317 (56)	73 (77)^b^	141 (69)^b^	103 (39)
Refractory fever	163 (29)	53 (57)^b,c^	68 (34)^b^	42 (16)
Recrudescent fever	18 (3)	4 (3)	7 (3)	7 (3)
Pleuritic chest pain	18 (3)	5 (5)	8 (4)	5 (2)
Pleuritic rub	6 (1)	3 (3)	0	3 (1)
Dyspnea	257 (46)	45 (48)^b,c^	129 (64)	83 (31)
Hemoptysis	32 (6)	14 (15)^b,c^	13 (6)	5 (2)
Worsening lung function	219 (39)	61 (66)^b^	118 (58)^b^	40 (15)
Abnormal radiologic findings	487 (87)	94 (100)^b^	203 (100)^b^	191 (71)
Chest X-ray/CT scan, *n* (%)	515 (91)/223 (40)	86 (92)/62 (67)^b,c^	192 (95)/96 (47)^b^	237 (89)/65 (24)
Non-specific chest CT scan findings, *n*	135/223	33/62^b^	55/96^b^	57/65
“Typical” chest CT scan findings, *n*	84/223	29/62^b^	41/96^b^	4/65
Microbiologic findings				
BAL^a^/ETA, *n* (%)	477 (96)	79 (92)	182 (98)	216 (97)
BAL^a^ performed, *n* (%)	225 (40)	56 (60)^b^	112 (55)^b^	57 (21)
Positive GM culture, *n*/mes (%)	86/151 (57)	44/52 (84)^b^	31/37 (84)^b^	11/62 (18)
β-d-glucan, *n*/mes (%)	3/6 (50)	0	3/5 (60)	0/1 (0)
PCR, *n*/mes (%)	4/4 (100)	0	3/3 (100)	1/1 (100)
Performed biopsy/autopsy, *n* (%)	72/61 (24)	93 (100)^b,c^	15 (7)	25 (9)
Isolated species, *n* (%)				
*Aspergillus fumigatus*	519 (92)	83 (88)	182 (90)	254 (96)
*Aspergillus flavus*	19 (3)	2 (2)	12 (6)	5 (2)
*Aspergillus niger*	7 (1)	2 (2)	2 (1)	3 (1)
Others	18 (3)	7 (8)	7 (3)	4 (2)
EORTC host factors, *n* (%)				
EORTC host factor present on diagnosis	249 (44)	65 (70)^b^	143 (70)^b^	41 (15)
Neutropenia	34 (6)	8 (9)^b^	21 (10)^b^	5 (2)
Malignancy under cytotoxic therapy	66 (12)	18 (19)^b^	37 (18)^b^	11 (4)
Glucocorticoid treatment	256 (45)	59 (63)^b,c^	156 (78)^b^	42 (15)
Inherited severe immunodeficiency	11 (2)	3 (3)	6 (3)	0
Organ support at time of diagnosis, *n* (%)				
Vasopressor therapy	346 (61)	63 (67)^b^	138 (68)^b^	145 (55)
RRT	155 (28)	48 (51)^b,c^	61 (30)^b^	46 (18)
Mechanical ventilation	482 (86)	92 (98)^b,c^	177 (87)	214 (80)
Organ support during ICU stay, *n* (%)				
Vasopressor therapy	429 (76)	78 (83)^b^	168 (83)^b^	183 (69)
RRT	182 (32)	57 (60)^b,c^	71 (35)^b^	54 (21)
Mechanical ventilation	506 (90)	86 (91)	187 (92)	233 (87)

^a^BAL, Bronchoalveolar lavage; CT, Computed tomography; EORTC, European Organization for Research and Treatment of Cancer; ETA, Endotracheal aspirate; GM, Galactomannan; IA, Invasive aspergillosis; ICU, Intensive care unit; RRT, Renal replacement therapy; SOFA, Sepsis Organ Failure Assessment. ^b^ *P* < 0.05 versus colonization; ^c^ *P* < 0.05 versus putative.

### Affected site and diagnostic classification

**Figure 1:**
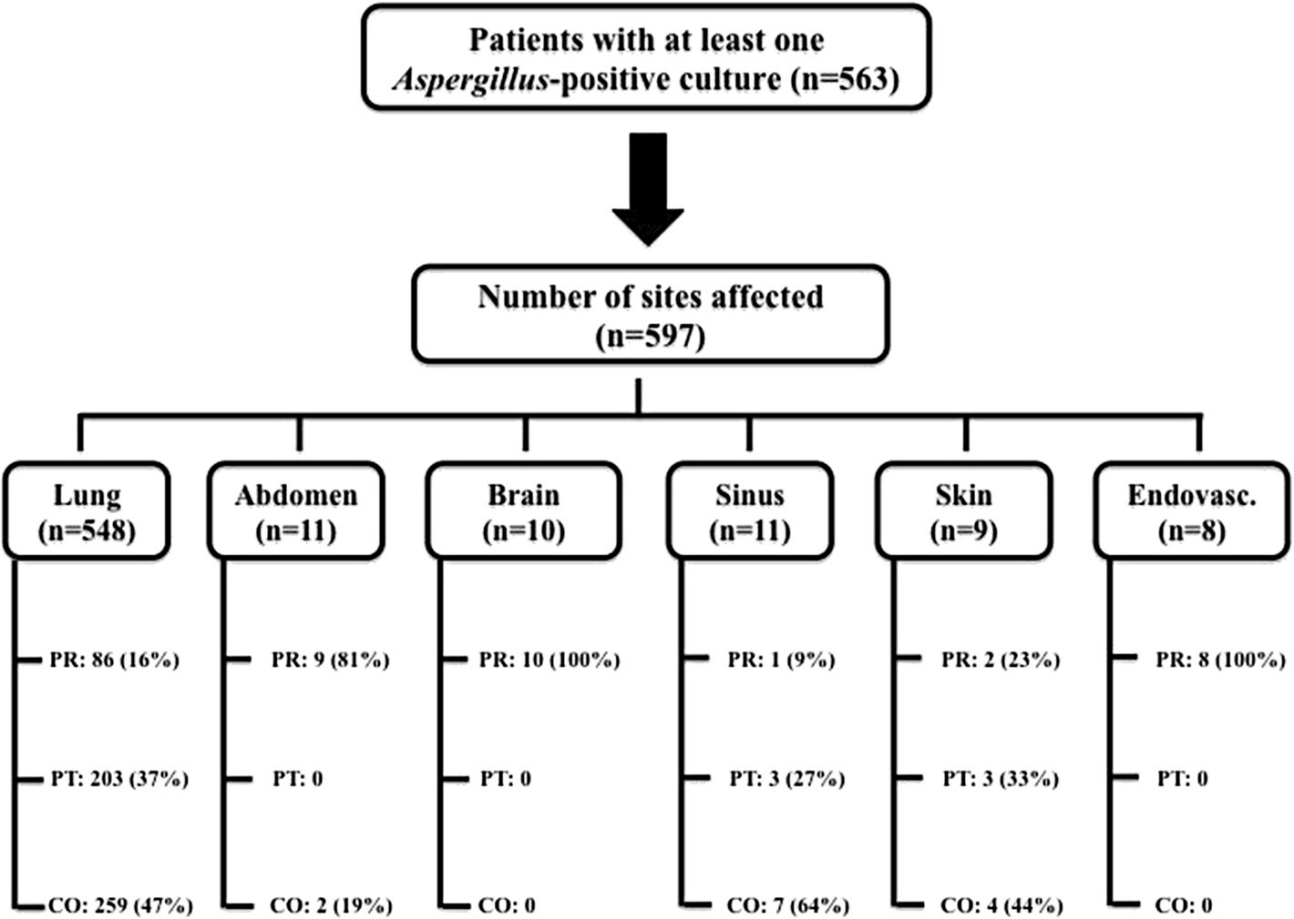
**Number of sites affected by** ***Aspergillus*** **spp for the different diagnostic categories.** Patients who had multiple sites positive for *Aspergillus* spp were counted more than once. CO, Colonization; PR, Proven; PT, Putative. Data are reported as number (%).

The most commonly affected sites were the lung and/or trachea (92%) (Figure 1). Only 16% of the patients with positive *Aspergillus* cultures in the lung and/or trachea had proven IA, whereas almost all patients with positive cultures in the abdominal, brain and endovascular samples were diagnosed with proven IA. Two positive abdominal samples were considered as colonization, as they originated from indwelling drains; the other nine patients had liver (*n* = 5) or peritoneal (*n* = 4) involvement. Twenty-eight patients had more than one site affected (twenty-two had two and six had three affected sites). Six patients with positive lung cultures were classified as “proven” because of non-pulmonary proven IA. Nine of ten patients with brain involvement and all eight with endovascular IA also had positive pulmonary cultures. Three of the patients with positive abdominal cultures (that is, proven disease) also had proven pulmonary IA. The seven patients with putative infection in the skin (*n* = 4) or the sinus (*n* = 3) were classified as such because of concomitant putative pulmonary localization; in particular, with the exception of one patient with proven disease, no specific skin biopsy was performed in any patient to confirm cutaneous aspergillosis.

Patients with putative or proven IA were more likely to have hematologic cancer and to have undergone bone marrow or solid organ transplant than were those with *Aspergillus* colonization, which likely explains the larger proportion of patients receiving chemotherapy, radiotherapy and immunosuppressive drugs in these groups. Patients with putative or proven IA were also more frequently admitted for a medical reason and were more likely to have a diagnosis of sepsis or ARDS on ICU admission. Although the radiologic findings were abnormal in all patients with proven or putative IA, no more than 40% had chest CT scan findings that were considered “typical” of IA. Where available serum or BAL GM measurements were available, abnormal results were present in more than 80% of patients with proven or putative IA, compared with only 18% of patients with colonization. Compared with colonized patients, patients with proven and putative IA had higher SOFA scores on the day of the first positive *Aspergillus* culture; these patients also had a greater requirement for mechanical ventilation, vasopressors or RRT during the ICU stay.

In patients with proven IA, COPD was observed less frequently than in patients with putative IA. In addition, compared with patients with putative IA, patients with a proven diagnosis were more frequently treated with immunosuppressive drugs, more frequently admitted for medical reasons, had a greater incidence of respiratory diseases and ARDS on ICU admission and higher SOFA scores on IA diagnosis. On the day of diagnosis, patients with proven IA had more compatible clinical signs (fever refractory to antibacterial therapy, dyspnea and hemoptysis) than those with putative IA, as well as more non-specific findings on CT scans. Most of the extrathoracic cases of *Aspergillus* were in patients with proven IA, on the basis of analysis of fluids obtained from sterile body sites or biopsies. RRT therapy was more commonly used among patients with proven IA. Antifungal therapy was initiated in 285 (51%) patients. In 31 additional cases, therapy was not administered on the basis of either a clinical decision or post-mortem diagnosis. The median time from positive *Aspergillus* culture to initiation of therapy was 2 (0 to 5) days for patients with *Aspergillus* colonization and 1 (0 to 3) days for both proven and putative IA.

### Immunocompromised patients

Among those patients with putative or proven IA, 208 (70%) were in an immunosuppressive state according to EORTC criteria (Table 3). Immunocompromised patients had severity of disease similar to others’ and presented more often with respiratory failure and less often with trauma as reasons for ICU admission. They also received prophylactic antifungal therapy more often than others and were less likely to be receiving RRT at IA diagnosis.

**Table 3:** **Main differences among patients with putative or proven invasive aspergillosis, with regard to presence of immunosuppression** ^**a**^

	**Immunosuppression (** ***n*** **= 208)**	**No immunosuppression (** ***n*** **= 89)**	***P*** **-value**
Age, yr	61 ± 15	63 ± 15	0.15
Male, *n* (%)	127 (61)	53 (60)	0.92
BMI, kg/m^2^	23 (20 to 27)	24 (21 to 28)	0.77
Underlying conditions, *n* (%)			
COPD	67 (32)	35 (39)	0.23
Chronic heart failure	14 (7)	13 (15)	0.06
Diabetes	28 (14)	24 (27)	0.02
Liver disease	16 (8)	11 (12)	0.19
Chronic hemodialysis	6 (3)	5 (6)	0.25
Smoking	24 (12)	19 (21)	0.03
Alcohol abuse	12 (6)	15 (17)	0.002
Diagnostic category, *n* (%)			
Medical admission	170 (82)	69 (78)	0.08
Cardiovascular	49 (24)	29 (33)	0.1
Respiratory	112 (54)	33 (37)	0.008
Neurological	4 (2)	2 (2)	0.85
Intoxication	1 (1)	0	0.51
Gastrointestinal	11 (5)	8 (9)	0.23
Endocrine	13 (6)	5 (6)	0.83
Post-operative	18 (9)	4 (4)	0.21
Trauma	0	5 (6)	0.006
Others	2 (1)	1 (1)	0.89
Severity scores and main diagnoses			
APACHE II score on admission	25 (17 to 30)	23 (17 to 28)	0.29
Sepsis on ICU admission	103 (50)	38 (42)	0.07
ARDS on ICU admission	33 (16)	22 (25)	0.07
Septic shock, *n* (%)	28 (13)	16 (18)	0.31
Traumatic brain injury, *n* (%)	1 (1)	0	0.51
Pneumonia, *n* (%)	27 (13)	17 (19)	0.17
Other brain injuries, *n* (%)	3 (1)	0	0.25
Acute heart failure/CABG, *n* (%)	1 (1)	8 (9)	<0.001
Pancreatitis, *n* (%)	2 (1)	0	0.35
Prophylactic antifungals, *n* (%)	24 (12)	3 (3)	0.03
Antifungal therapy, *n* (%)	159 (76)	66 (74)	0.67
Time from diagnosis to therapy, days	1 (0 to 3)	1 (0 to 4)	0.34
Duration of therapy, days^b^	11 (6 to 25)	14 (5 to 28)	0.83
ICU stay before first positive culture, days	4 (2 to 11)	4 (2 to 10)	0.76
SOFA II score at diagnosis	10 (6 to 13)	9 (6 to 12)	0.15
Organ support at time of diagnosis, *n* (%)			
Vasopressor therapy	139 (67)	62 (70)	0.63
RRT	67 (32)	42 (47)	0.01
Mechanical ventilation	188 (90)	81 (91)	0.87
Organ support during ICU stay, *n* (%)			
Vasopressor therapy	173 (83)	73 (82)	0.81
RRT	83 (40)	45 (51)	0.09
Mechanical ventilation	190 (91)	83 (93)	0.58
12-week mortality	151 (73)	59 (66)	0.27

^a^ARDS, Acute respiratory distress syndrome; APACHE, Acute Physiology and Chronic Health Evaluation; BMI, Body mass index; CABG, Coronary artery bypass graft; COPD, Chronic obstructive pulmonary disease; ICU, Intensive care unit; RRT, Renal replacement therapy; SOFA, Sequential Organ Failure Assessment. ^b^Calculated among those patients receiving therapy.

### Outcomes

The length of ICU stay was similar in patients with proven IA, putative IA and *Aspergillus* colonization (median 15 days [1st-3rd quartile, 8-32 days] vs. 17 days [1st-3rd quartile, 9-35 days] vs. 14 days [1st-3rd quartile, 6-30 days]; p=0.07). Mortality at 12 weeks was significantly higher in patients with proven IA (74 of 94, 79%) than in those with putative IA (136 of 203, 67%) (*P* = 0.03) or colonization (101 of 266, 38%) (*P* < 0.001). Patients with pulmonary involvement had mortality rates of 55% (71% if proven IA, 68% if putative IA and 39% if *Aspergillus* colonization), which were similar to the rates in patients with skin and/or wound and sinus involvement (55% and 37%, respectively). The highest mortality rates were observed in patients with abdominal (81%), cerebral (90%) or endovascular (86%) involvement. Among patients with putative or proven IA, mortality was similar between those who met EORTC criteria for immunosuppression and others (73% versus 66%, *P* = 0.27). No differences in overall survival were observed when patients who received antifungal therapy were compared with untreated patients. Also, the delay between the first positive *Aspergillus* culture and the initiation of antifungal therapy was not associated with outcome.

**Figure 2:**
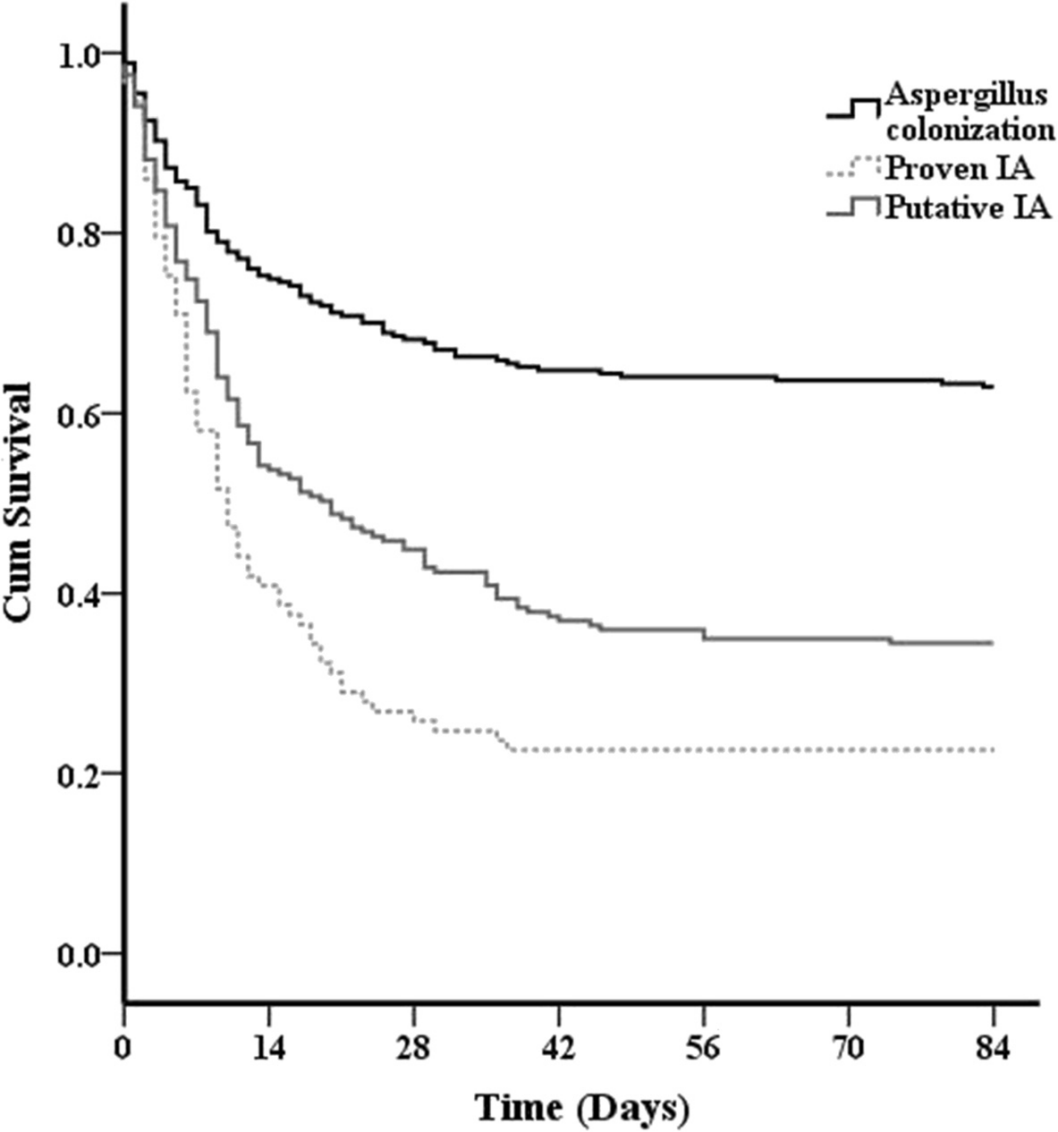
**Survival curves for different diagnostic categories using the criteria of the clinical algorithm.** The result of the log-rank analysis for survival distributions was *P* < 0.001 when putative or proven invasive aspergillosis (IA) was compared with colonization. The result of the log-rank analysis for survival distribution between putative and proven IA was *P* = 0.156. See Appendix 2 for clinical algorithm.

At 84 days, patients with putative and proven IA had significantly lower survival rates than those with colonization (log-rank *P* < 0.001) (Figure 2). After adjustment for several confounders, this difference remained significant only for proven IA (Table 4). Among patients with proven or putative IA, independent risk factors for mortality were older age, bone marrow transplant, higher SOFA score and need for mechanical ventilation or RRT on the day of positive *Aspergillus* culture (Table 5).

**Table 4:** **Mortality risk of putative and proven invasive aspergillosis relative to** ***Aspergillus*** **colonization** ^**a**^

	**Unadjusted**				**Adjusted**			
		**95% CI for HR**				**95% CI for HR**		
	**HR**	**Lower**	**Upper**	***P*** **-value**	**HR**	**Lower**	**Upper**	***P*** **-value**
Colonization	1.00				1.00			
Putative IA	2.18	1.68	2.83	< 0.001	1.18	0.93	1.81	0.16
Proven IA	3.12	2.30	4.24	< 0.001	1.51	1.00	2.26	0.05

^a^CI, Confidence interval; HR, Hazard ratio; IA, Invasive aspergillosis.

**Table 5:** **Risk factors for mortality among patients with proven or putative invasive aspergillosis**

**Variable**	**Univariable analysis**		**Multivariable analysis**	
	***P*** **-value**	**OR (95% CI)**	***P***-**value**	**OR (95% CI)**
Age, yr	0.008	1.023 (1.006 to 1.040)	0.001	1.034 (1.014 to 1.055)
Male	0.264	0.751 (0.455 to 1.241)		
BMI	0.024	1.069 (1.009 to 1.133)		
Septic shock	0.689	1.158 (0.565 to 2.374)		
Pneumonia	0.880	0.948 (0.476 to 1.888)		
Primary brain injury	0.216	0.218 (0.020 to 2.437)		
Acute cardiac failure	0.225	3.655 (0.450 to 29.660)		
Sepsis on admission	0.110	1.503 (0.912 to 2.478)		
APACHE^a^ II score at admission	0.003	1.049 (1.017 to 1.083)		
Diabetes	0.576	1.211 (0.619 to 2.371)		
Chronic heart disease	0.030	3.890 (1.140 to 13.266)		
COPD	0.605	0.872 (0.520 to 1.463)		
Liver failure	0.070	2.749 (0.922 to 8.192)		
HIV	0.564	0.441 (0.027 to 7.132)		
Smoking	0.937	1.029 (0.509 to 2.078)		
Alcohol abuse	0.298	0.639 (0.276 to 1.483)		
Chronic dialysis	0.148	4.615 (0.582 to 36.603)		
Bone marrow transplant	0.013	3.875 (1.326 to 11.327)	0.039	3.352 (1.060 to 10.598)
Solid tumor	0.085	2.241 (0.894 to 5.617)		
Cancer	0.769	1.101 (0.579 to 2.094)		
Neutropenia	0.646	0.827 (0.368 to 1.858)		
Chemotherapy/radiotherapy	0.525	0.802 (0.405 to 1.586)		
Solid organ transplant	0.877	1.055 (0.534 to 2.084)		
Corticosteroids	0.248	1.376 (0.801 to 2.366)		
Immune deficit	0.117	0.342 (0.090 to 1.306)		
*Aspergillus* species	0.040	2.183 (1.038 to 4.592)		
Lung involvement	0.998	1.001 (0.300 to 3.340)		
SOFA score at diagnosis	<0.001	1.180 (1.107 to 1.256)	<0.001	1.140 (1.062 to 1.224)
Vasopressor therapy at diagnosis	<0.001	4.309 (2.299 to 8.078)		
Mechanical ventilation at diagnosis	<0.001	6.498 (2.590 to 16.303)	0.009	3.916 (1.408 to 10.891)
Renal replacement therapy at diagnosis	<0.001	3.293 (1.895 to 5.722)	0.011	2.339 (1.212 to 4.516)

^a^APACHE, Acute Physiology and Chronic Health Evaluation; BMI, Body mass index; CI, Confidence interval; COPD, Chronic obstructive pulmonary disease; IA, Invasive aspergillosis; OR, Odds ratio; RRT, Renal replacement therapy; SOFA, Sequential Organ Failure Assessment.

## Discussion

In this cohort of 563 patients with an *Aspergillus*-positive cultures, 53% were diagnosed with putative or proven IA. Compared with patients with *Aspergillus* colonization, more patients with IA had cancer, organ transplant or sepsis and/or ARDS on admission, and more needed supportive therapy. They also had higher mortality. Patients with proven IA had a greater incidence of medical admissions and higher SOFA scores than patients with putative IA, but the outcomes in the groups were similar. Older age, bone marrow transplant, higher SOFA score and mechanical ventilation or RRT were independently associated with poor outcome.

The true epidemiology of IA remains uncertain and depends on case mix, environmental factors and diagnostic strategy. In our cohort, clinical and radiologic manifestations were non-specific and may have been masked by the underlying acute process. Although Barberan *et al*. showed that radiologic worsening or cavitation visualized on chest X-rays and/or CT scans was associated with IA [30], in another series up to 60% of cases of IA were exclusively diagnosed by autopsy because of lack of reliable clinical and radiologic tests [7]. Moreover, in an ICU cohort, only 12 of 67 ICU patients with proven IA had a halo sign or cavitation visualized on chest CT scans [14]. To overcome these limitations, the use of BAL culture for GM has been suggested, which, with a cutoff of 0.5, was found to have 88% sensitivity and 87% specificity to detect IA, whereas the sensitivity of serum GM was only 42% [5]. In our study, we found a higher proportion of proven and putative IA cases with positive GM levels (both 84%), as compared with only 18% in patients with *Aspergillus* colonization. However, with GM in ICU patients being limited to suspected cases [6,30], use of a clinical algorithm to discriminate colonization from IA [23] remains a valid option for identifying patients who require a more extensive workup.

Few studies have been done on the epidemiology of IA in ICUs. In a large US cohort, ICU patients with aspergillosis were found to have several comorbid diseases, high mortality, prolonged hospitalization and increased costs [18]. Specifically, over 70% of patients needed ventilation and received high-dose corticosteroids, and over 35% had acute renal failure, COPD or septic shock. In a multicenter Italian study, aspergillosis represented 35 of 384 invasive mycoses in ICU patients [31]. Previous corticosteroid administration for autoimmune disease or COPD was the major host factor associated with IA. IA occurred more frequently in medical patients, and mortality attributed to aspergillosis was higher compared with candidiasis (63% versus 46%; *P* = 0.01). In these studies, researchers considered only possible and proven IA, however. Petri *et al*. isolated *Aspergillus* in only 4% of 435 non-neutropenic ICU patients, but none had IA [32]. In contrast, in the present study, *Aspergillus* isolation appeared to represent IA in over 50% of patients. Immunosuppression and sepsis and/or ARDS at ICU admission were more frequent in patients with proven or putative IA than in patients with *Aspergillus* colonization. A particular feature of the present study was the finding that an alternative diagnostic algorithm in patients with *Aspergillus*-positive respiratory tract cultures encompassed a larger segment of the burden of IA than the stricter EORTC/MSG criteria [9]. Hence, identification of risk factors for IA may help determine which ICU patients would benefit from specific screening when an *Aspergillus*-positive culture is found. Another new finding is that we could describe the epidemiology of non-pulmonary *Aspergillus* cultures in ICU patients, which were predominantly associated with proven IA, as affected organs were frequently considered sterile body sites, such as the brain [33].

Pulmonary IA has been documented in 15% of bone marrow transplant patients [34]. Prolonged neutropenia and solid organ transplant also have been identified as high-risk factors [34-37]. These conditions are rare in ICU patients. In one study, only 14% of ICU patients with IA were neutropenic [7]. Risk factors for IA in ICU patients include COPD, liver cirrhosis and severe sepsis [38,39]. In patients with COPD, the incidence of *Aspergillus* isolates from lower respiratory tract samples has increased progressively [40]. In a study of 118 patients with COPD, 60% were colonized and 40% had IA [41]. The patients with IA in that study were more likely to have advanced respiratory disease, as suggested by the Global initiative for Obstructive Lung Disease classification. The patients with IA had higher severity scores and had worse prognoses than patients with *Aspergillus* colonization. The emergence of IA in patients with COPD is attributed mainly to prolonged administration of corticosteroids [42]. Corticosteroids also represent a risk for IA in ICU patients receiving immunosuppression following solid organ transplant or for autoimmune disease [14]. Corticosteroids predispose patients to opportunistic infections through quantitative and qualitative functional impairment of macrophages and neutrophil function [42]. Liver cirrhosis also unfavorably alters humoral and cellular immune response or complement activity, thereby increasing the risk for infections [43]. Sepsis-associated immunoregulatory disturbances, such as macrophage deactivation and altered cellular immune response, can induce a state of immunoparalysis, hampering adequate host response to fungal disease [44]. Recently, IA was also associated with H1N1 infection, suggesting that viral infections may play a causal role [45]. Unfortunately, we did not collect data on H1N1 co-infection. We also found that higher SOFA scores and RRT requirement were associated with IA.

We report a 72% 12-week mortality rate in patients with IA in the present study. In a previous study on medical ICU patients, mortality was 92% [46]; however, most of those patients had hematologic disease or neutropenia, which may have influenced their survival. In our study, a minority of patients had cancer and/or were receiving immunosuppressive therapy. Our findings also provide interesting data on a general ICU population. Mortality rates have improved in ICU patients who develop IA. In a recent study focused on hospitalized patients with IA, investigators reported a crude mortality rate of 37% [47]. In another study, mortality for IA was 50% [6]. However, because IA often develops in patients with greater disease severity, it remains difficult to determine whether this fungal disease contributes *per se* to the poor outcome or if it is just a marker of disease severity. Khasawneh *et al*. reported a 15% greater mortality among ICU patients with IA than that predicted by APACHE II score [6]. Those authors also showed similar mortality rates in patients with colonization and those with invasive disease. In another study, researchers estimated the attributable mortality for IA to be 19% [48]. Both studies [6,48] were biased by a lack of a diagnostic approach to differentiate colonization from IA. Also, the availability of more effective antifungal agents in the later study [48] may explain the differences. In our present study, patients with putative and proven IA had significantly higher mortality rates than patients with *Aspergillus* colonization; however, after adjustment for several confounders, this difference remained significant only for patients with proven IA. Older age, bone marrow transplant, higher SOFA score and/or need for mechanical ventilation or RRT predicted poor outcome in IA. These findings highlight the need for a more timely diagnostic approach to evaluate the effect of earlier initiation of therapy.

Our study has some limitations. First, we could not analyze a specific multimodal diagnostic approach, as only a minority of patients had CT scans and GM measurements. Moreover, we did not specifically collect whether GM measurement was performed in blood, BAL or other biological fluid samples. The rare use of GM in the diagnostic algorithm could be related to the cost of the analysis and/or to its delayed implementation, as the present study was initiated before the publication of the most important studies dealing with GM assessment in critically ill patients [5,22]. Additionally, we included only patients with *Aspergillus*-positive cultures, thereby excluding patients with suspected disease based on radiologic imaging or biomarkers. Second, we did not assess reasons for death and cannot exclude the possibility that some patients died because of concomitant conditions. Third, we did not collect any data on the incidence of positive *Aspergillus* cultures or the total number of ICU admissions to the participating centers over the study period. Thus, we could not evaluate the burden of this disease among critically ill patients. Importantly, IA remains a rare disease. It was reported in 1% to 2% of ICU patients in a large international database [10]. Fourth, we did not record laboratory data in detail, which might have been useful to better characterize the cohort. We also did not collect data on daily corticosteroid dose and could not fully explore the impact of dose or duration on mortality. Importantly, we did not collect the amount of antibacterial drugs that patients received before IA diagnosis or whether this may have influenced the development of the disease. Fifth, routine autopsy would have increased the number of cases of IA diagnosis. Autopsy studies have shown that IA is the most frequently missed infection diagnosis in patients requiring ICU admission [15,49,50]. Sixth, we did not consider environmental sources of IA, such as construction work contaminating hospital air [51,52]. Seventh, some of these data were reported in a previous publication; however, we focused on epidemiologic findings, whereas the previous study aimed to validate the diagnostic accuracy of a clinical algorithm. Eighth, although the drug of choice for patients who do not have neutropenia needs to be further studied, we do not report data on the differences between the drugs used to treat *Aspergillus* spp in this cohort [53].

## Conclusions

Compared with patients with *Aspergillus* colonization, patients with IA more frequently had sepsis or respiratory failure at the time of admission and had more underlying conditions, including immunocompromised status. Patients with IA had higher disease severity and needed more organ support than patients with *Aspergillus* colonization. IA in the ICU is associated with an unacceptably high mortality rate.

## Key messages

- In this multicentric cohort of ICU patients, half of the patients with a positive *Aspergillus* culture had either putative or proven IA.
- Patients with IA were more frequently immunosuppressed than those with *Aspergillus* colonization; however, they also more frequently had sepsis and higher severity of illness.
- Mortality in patients with IA was significantly higher than in those with *Aspergillus* colonization, even after adjustment for several risk factors.

## Appendix 1

The following are the ethics committees and institutional review boards of the collaborating centers:

- Comité Ético de Investigación Clínica, Hospital Universitario Severo Ochoa (Madrid, Spain)
- Comité d’Ethique Médicale, Cliniques Universitaires UCL de Mont-Godinne (Yvoir, Belgium)
- Comissão de Ética, Hospital de Santo Antonio (Porto, Portugal)
- Commission d’Ethique de la Société de Réanimation de Langue Francaise (France)
- Comité d’Ethique, Cliniques de l’Europe (Brussels, Belgium)
- Comité d’Ethique, Cliniques Universitaires Saint Luc (Brussels, Belgium)
- Medisch Ethische Commissie, H-Hartziekenhuis Roeselare-Menen (Roeselare, Belgium)
- Ethics Committee of University Hospital Attikon (Athens, Greece)
- Comité d’Ethique, Centre Hôspitalier Régional Mons-Warquignies (Mons, Belgium)
- Research Ethics Committee, Shanghai Public Health Clinical Center, Fudan University (Shangai, China)
- Comité d’Ethique, Centre Hospitalier du Grand Hornu (Hornu, Belgium)
- Commissie Medische Ethiek, UZ Leuven (Leuven, Belgium)
- Comissão de Ética, Hospital de Sao Joao (Porto, Portugal)
- Comissão de Ética do Santa Casa-Complexo Hospitalar (Porto Alegre, Brazil)
- Comité Ético de Investigación Clínica, Vall d’Hebron University Hospital (Barcelona, Spain)
- Ethics Committee, Apollo Hospital (Jubilee Hills, Hyderabad, India)
- Comitè d’Ètica d’Investigació Clínica, Hospital Universitari de Tarragona Joan XXIII (Tarragona, Spain)
- Commissie Medische Ethiek, Reflectiegroep Biomedische Ethiek, Universitair Ziekenhuis Brussel (Brussels, Belgium)
- Comité d’Ethique, Hôpital Erasme (Brussels, Belgium)
- Commissie Medische Ethiek, AZ St Lucas (Ghent, Belgium)
- Ethisch Comité, UZ Gent (Ghent, Belgium)

## Appendix 2

The text in this appendix describes the clinical algorithm used to diagnose invasive pulmonary aspergillosis in critically ill patients [23].

### Proven invasive pulmonary aspergillosis

Proven invasive pulmonary aspergillosis is determined by microscopic analysis of sterile material in which histopathologic, cytopathologic or direct microscopic examination of a specimen obtained by needle aspiration or sterile biopsy reveals hyphae accompanied by evidence of associated tissue damage; or, an *Aspergillus*-positive culture on sterile material obtained by lung biopsy.

### Putative invasive pulmonary aspergillosis (all four criteria must be met)

1. *Aspergillus*-positive lower respiratory tract specimen culture (entry criterion)
2. Compatible signs and symptoms (one of the following):
  - Fever refractory to at least 3 days of appropriate antibiotic therapy
  - Recrudescent fever after a period of defervescence of at least 48 hours while still on antibiotics and without other apparent cause
  - Pleuritic chest pain
  - Pleuritic rub
  - Dyspnea
  - Hemoptysis
  - Worsening respiratory insufficiency in spite of appropriate antibiotic therapy and ventilatory support
3. Abnormal medical imaging by portable chest X-ray or CT scan of the lungs
4. Either or both of the following:
  - Host risk factors (one of the following conditions):
    i. Neutropenia (absolute neutrophil count <500/mm^3^) preceding or at the time of ICU admission
    ii. Underlying hematological or oncological malignancy treated with cytotoxic agents
    iii. Glucocorticoid treatment (prednisone equivalent >20 mg/day)
    iv. Congenital or acquired immunodeficiency
  - Semiquantitative *Aspergillus*-positive culture of BAL fluid (+ or ++) without bacterial growth, together with a positive cytological smear showing branching hyphae

### Respiratory tract *Aspergillus* colonization

When more than one criterion necessary for a diagnosis of putative IPA is not met, the case is classified as *Aspergillus* colonization.

## Abbreviations

APACHE: Acute Physiology and Chronic Health Evaluation
ARDS: Acute respiratory distress syndrome
BAL: Bronchoalveolar lavage
BMI: Body mass index
BMT: Bone marrow transplant
CABG: Coronary bypass graft
CI: Confidence interval
COPD: Chronic obstructive pulmonary disease
CSF: Cerebrospinal fluid
CT: Computed tomography
EORTC: European Organization for Research and Treatment of Cancer
ETA: Endotracheal aspirate
GM: Galactomannan
HR: Hazard ratio
IA: Invasive aspergillosis
ICU: Intensive care unit
MSG: Mycosis Study Group
OR: Odds ratio
RRT: Replacement therapy
SOFA: Sequential Organ Failure Assessment

## References

[1] Meersseman W, Van Wijngaerden E (2007). Invasive aspergillosis in the ICU: an emerging disease. Intensive Care Med..

[2] Auberger J, Lass-Flörl C, Ulmer H, Nogler-Semenitz E, Clausen J, Gunsilius E (2008). Significant alterations in the epidemiology and treatment outcome of invasive fungal infections in patients with hematological malignancies. Int J Hematol..

[3] Eriksson M, Lemström K, Suojaranta-Ylinen R, Martelius T, Harjula A, Sipponen J (2010). Control of early *Aspergillus* mortality after lung transplantation: outcome and risk factors. Transplant Proc..

[4] Lugosi M, Alberti C, Zahar JR, Garrouste M, Lemiale V, Descorps-Desclère A (2014). *Aspergillus* in the lower respiratory tract of immunocompetent critically ill patients. J Infect..

[5] Meersseman W, Lagrou K, Maertens J, Wilmer A, Hermans G, Vanderschueren S (2008). Galactomannan in bronchoalveolar lavage fluid: a tool for diagnosing aspergillosis in intensive care unit patients. Am J Respir Crit Care Med..

[6] Khasawneh F, Mohamad T, Moughrabieh MK, Lai Z, Ager J, Soubani AO (2006). Isolation of *Aspergillus* in critically ill patients: a potential marker of poor outcome. J Crit Care..

[7] Garnacho-Montero J, Amaya-Villar R, Ortiz-Leyba C, León C, Alvarez-Lerma F, Nolla-Salas J (2005). Isolation of *Aspergillus* spp. from the respiratory tract in critically ill patients: risk factors, clinical presentation and outcome. Crit Care.

[8] Vandewoude K, Blot S, Benoit D, Depuydt P, Vogelaers D, Colardyn F (2004). Invasive aspergillosis in critically ill patients: analysis of risk factors for acquisition and mortality. Acta Clin Belg..

[9] De Pauw B, Walsh TJ, Donnelly JP, Stevens DA, Edwards JE, Calandra T (2008). Revised definitions of invasive fungal disease from the European Organization for Research and Treatment of Cancer/Invasive Fungal Infections Cooperative Group and the National Institute of Allergy and Infectious Diseases Mycoses Study Group (EORTC/MSG) Consensus Group. Clin Infect Dis..

[10] Vincent JL, Sakr Y, Sprung CL, Ranieri VM, Reinhart K, Gerlach H (2006). Sepsis in European intensive care units: results of the SOAP study. Crit Care Med..

[11] Petri MG, König J, Moecke HP, Gramm HJ, Barkow H, Kujath P (1997). Paul-Ehrlich Society for Chemotherapy, Divisions of Mycology and Pneumonia Research. Epidemiology of invasive mycosis in ICU patients: a prospective multicenter study in 435 non-neutropenic patients. Intensive Care Med.

[12] Blot S, Charles PE (2013). Fungal sepsis in the ICU: are we doing better? Trends in incidence, diagnosis, and outcome. Minerva Anestesiol..

[13] Montagna MT, Caggiano G, Lovero G, De Giglio O, Coretti C, Cuna T (2014). Epidemiology of invasive fungal infections in the intensive care unit: results of a multicenter Italian survey (AURORA Project). Infection..

[14] Meersseman W, Vandecasteele SJ, Wilmer A, Verbeken E, Peetermans WE, Van Wijngaerden E (2004). Invasive aspergillosis in critically ill patients without malignancy. Am J Respir Crit Care Med..

[15] Dimopoulos G, Piagnerelli M, Berré J, Eddafali B, Salmon I, Vincent JL (2003). Disseminated aspergillosis in intensive care unit patients: an autopsy study. J Chemother..

[16] Vandewoude KH, Blot SI, Depuydt P, Benoit D, Temmerman W, Colardyn F (2006). Clinical relevance of *Aspergillus* isolation from respiratory tract samples in critically ill patients. Crit Care..

[17] Levy H, Horak DA, Tegtmeier BR, Yokota SB, Forman SJ (1992). The value of bronchoalveolar lavage and bronchial washings in the diagnosis of invasive pulmonary aspergillosis. Respir Med..

[18] Baddley JW, Stephens JM, Ji X, Gao X, Schlamm HT, Tarallo M (2013). Aspergillosis in Intensive Care Unit (ICU) patients: epidemiology and economic outcomes. BMC Infect Dis..

[19] Donati DY, Papazian L (2008). Role of open-lung biopsy in acute respiratory distress syndrome. Curr Opin Crit Care..

[20] Koulenti D, Garnacho-Montero J, Blot S (2014). Approach to invasive pulmonary aspergillosis in critically ill patients. Curr Opin Infect Dis..

[21] Koulenti D, Vogelaers D, Blot S (2014). What’s new in invasive pulmonary aspergillosis in the critically ill?. Intensive Care Med..

[22] He H, Ding L, Chang S, Li F, Zhan Q (2011). Value of consecutive galactomannan determinations for the diagnosis and prognosis of invasive pulmonary aspergillosis in critically ill chronic obstructive pulmonary disease. Med Mycol..

[23] Blot SI, Taccone FS, Van den Abeele AM, Bulpa P, Meersseman W, Brusselaers N (2012). A clinical algorithm to diagnose invasive pulmonary aspergillosis in critically ill patients. Am J Respir Crit Care Med..

[24] AspICU2 Project. http://www.aspicu2.org/. Accessed 31 January 2015.

[25] Knaus WA, Draper EA, Wagner DP, Zimmerman JE (1985). APACHE II: a severity of disease classification system. Crit Care Med..

[26] Vincent JL, Moreno R, Takala J, Willatts S, De Mendonça A, Bruining H (1996). on behalf of the Working Group on Sepsis-Related Problems of the European Society of Intensive Care Medicine. The SOFA (Sepsis-related Organ Failure Assessment) score to describe organ dysfunction/failure. Intensive Care Med.

[27] Bernard GR, Artigas A, Brigham KL, Carlet J, Falke K, Hudson L (1994). The American-European Consensus Conference on ARDS. Definitions, mechanisms, relevant outcomes, and clinical trial coordination. Am J Respir Crit Care Med.

[28] Levy MM, Fink MP, Marshall JC, Abraham E, Angus D, Cook D (2003). 2001 SCCM/ESICM/ACCP/ATS/SIS International Sepsis Definitions Conference. Crit Care Med..

[29] Ascioglu S, Rex JH, de Pauw B, Bennett JE, Bille J, Crokaert F (2002). Defining opportunistic invasive fungal infections in immunocompromised patients with cancer and hematopoietic stem cell transplants: an international consensus. Clin Infect Dis..

[30] Barberan J, Alcazar B, Malmierca E (2012). Garcia de la Llana F, Dorca J, del Castillo D, et al. Repeated *Aspergillus* isolation in respiratory samples from non-immunocompromised patients not selected based on clinical diagnoses: colonisation or infection?. BMC Infect Dis..

[31] Tortorano AM, Dho G, Prigitano A, Breda G, Grancini A, Emmi V (2012). Invasive fungal infections in the intensive care unit: a multicentre, prospective, observational study in Italy (2006–2008). Mycoses..

[32] Petri MG, König J, Moecke HP, Gramm HJ, Barkow H, Kujath P (1997). Epidemiology of invasive mycosis in ICU patients: a prospective multicenter study in 435 non-neutropenic patients. Intensive Care Med..

[33] Spapen H, Spapen J, Taccone FS, Meersseman W, Rello J, Dimopoulos G (2014). Cerebral aspergillosis in adult critically ill patients: a descriptive report of 10 patients from the *Asp*ICU cohort. Int J Antimicrob Agents..

[34] Baddley JW, Stroud TP, Salzman D, Pappas PG (2001). Invasive mold infections in allogeneic bone marrow transplant recipients. Clin Infect Dis..

[35] Baddley JW, Andes DR, Marr KA, Kontoyiannis DP, Alexander BD, Kauffman CA (2010). Factors associated with mortality in transplant patients with invasive aspergillosis. Clin Infect Dis..

[36] Bouza E, Guinea J, Peláez T, Pérez-Molina J, Alcalá L, Muñoz P (2005). Workload due to *Aspergillus fumigatus* and significance of the organism in the microbiology laboratory of a general hospital. J Clin Microbiol..

[37] Burghi G, Lemiale V, Seguin A, Lambert J, Lacroix C, Canet E (2011). Outcomes of mechanically ventilated hematology patients with invasive pulmonary aspergillosis. Intensive Care Med..

[38] Bulpa PA, Dive AM, Garrino MG, Delos MA, Gonzalez MR, Evrard PA (2001). Chronic obstructive pulmonary disease patients with invasive pulmonary aspergillosis: benefits of intensive care?. Intensive Care Med..

[39] Gustot T, Maillart E, Bocci M, Surin R, Trépo E, Degré D (2014). Invasive aspergillosis in patients with severe alcoholic hepatitis. J Hepatol..

[40] Guinea J, Torres-Narbona M, Gijón P, Muñoz P, Pozo F, Peláez T (2010). Pulmonary aspergillosis in patients with chronic obstructive pulmonary disease: incidence, risk factors, and outcome. Clin Microbiol Infect..

[41] Barberan J, Sanz F, Hernandez JL, Merlos S, Malmierca E, Garcia-Perez FJ (2012). Clinical features of invasive pulmonary aspergillosis vs. colonization in COPD patients distributed by GOLD stage. J Infect.

[42] Ader F, Nseir S, Le Berre R, Leroy S, Tillie-Leblond I, Marquette CH (2005). Invasive pulmonary aspergillosis in chronic obstructive pulmonary disease: an emerging fungal pathogen. Clin Microbiol Infect..

[43] Marr KA, Carter RA, Crippa F, Wald A, Corey L (2002). Epidemiology and outcome of mould infections in hematopoietic stem cell transplant recipients. Clin Infect Dis..

[44] Falcone M, Massetti AP, Russo A, Vullo V, Venditti M (2011). Invasive aspergillosis in patients with liver disease. Med Mycol..

[45] Hartemink KJ, Paul MA, Spijkstra JJ, Girbes AR, Polderman KH (2003). Immunoparalysis as a cause for invasive aspergillosis?. Intensive Care Med..

[46] Wauters J, Baar I, Meersseman P, Meersseman W, Dams K, De Paep R (2012). Invasive pulmonary aspergillosis is a frequent complication of critically ill H1N1 patients: a retrospective study. Intensive Care Med..

[47] Janssen JJ (1996). Strack van Schijndel RJ, van der Poest Clement EH, Ossenkoppele GJ, Thijs LG. Huijgens PC. Outcome of ICU treatment in invasive aspergillosis. Intensive Care Med..

[48] Kim A, Nicolau DP, Kuti JL (2011). Hospital costs and outcomes among intravenous antifungal therapies for patients with invasive aspergillosis in the United States. Mycoses..

[49] Vandewoude KH, Blot SI, Benoit D, Colardyn F, Vogelaers D (2004). Invasive aspergillosis in critically ill patients: attributable mortality and excesses in length of ICU stay and ventilator dependence. J Hosp Infect..

[50] Robinett KS, Weiler B, Verceles AC (2013). Invasive aspergillosis masquerading as catastrophic antiphospholipid syndrome. Am J Crit Care..

[51] Winters B, Custer J, Galvagno SM, Colantuoni E, Kapoor SG, Lee H (2012). Diagnostic errors in the intensive care unit: a systematic review of autopsy studies. BMJ Qual Saf..

[52] Mullins J, Harvey R, Seaton A (1976). Sources and incidence of airborne *Aspergillus fumigatus* (Fres). Clin Allergy..

[53] Peláez T, Muñoz P, Guinea J, Valerio M, Giannella M, Klaassen CHW (2012). Outbreak of invasive aspergillosis after major heart surgery caused by spores in the air of the intensive care unit. Clin Infect Dis..

